# A Prospective Comparative Study for the Evaluation of Digital Learning as an Academic Tool for MBBS Phase III Students

**DOI:** 10.7759/cureus.111883

**Published:** 2026-07-01

**Authors:** Lavanya Anuranjani, Aalok Kumar, Ayub Khan

**Affiliations:** 1 Obstetrics and Gynaecology, Institute of Medical Sciences, Banaras Hindu University, Varanasi, IND; 2 Ophthalmology, Institute of Medical Sciences, Banaras Hindu University, Varanasi, IND; 3 Community Medicine, Institute of Medical Sciences, Banaras Hindu University, Varanasi, IND

**Keywords:** digital learning, e learning, learning management systems (lms), medical education (med ed) learning classroom integrated, phase 3 mbbs students, traditional learning

## Abstract

Introduction: This project seeks to redefine teaching and learning methods for MBBS students by integrating digital learning as an academic tool. The primary objective of this study was to enhance revision and promote audiovisual learning among students to improve their comprehension and understanding of the subject matter.

Materials and methods: The study involved 120 students who attended a 90-minute traditional lecture, delivered in two sessions, on a specific competency "Labour". Labour was chosen as it was a part of the core competency according to the NMC (National Medical Commission) curriculum. Following the lecture, a pre-test consisting of 20 multiple-choice questions (MCQs) was administered via Google Forms to assess knowledge acquisition after the traditional learning session. Subsequently, the students were divided into two groups of 60 each based on their odd- or even-numbered roll numbers assigned by the university. The roll numbers of students were allotted as per their names in alphabetical order; roll numbers had no relation to the academic performance of the students. One group received a digital learning module designed as a visual escape room, with quiz-mode MCQs administered via Google Forms. Students advanced to the next question only upon correctly answering the previous one, and additional information on the topic was provided for each correct response. The other group continued their learning traditionally. A second assessment was conducted one month later for all students using a post-test comprising 20 MCQs. The pre- and post-test scores of both groups were then analysed statistically. An unpaired Student's t-test was employed to analyse the results. A p-value < 0.05 was considered statistically significant.

Results: The group that followed the traditional teaching method achieved an average score of 15.9 out of 20, while the group utilising digital learning tools attained an average score of 17.3 out of 20. The increase in mean scores for the group using digital learning tools was statistically significant (p=0.002). Feedback from the digital group indicated that the digital learning approach was both acceptable and useful to students. These findings suggest that such modules can also serve as effective tools for formative assessment.

Conclusion: This study highlights the importance of innovative methods in facilitating repeated revision and improving recall. Given the extensive medical curriculum, limited time is allocated for effective student revision. The digital module supplements the learning experience and can be tailored to meet the needs of both students and teachers, providing a personalised, adaptable approach that can be repeated across various question types to address multiple domains of learning. A combination of traditional teaching and digital learning may yield better outcomes, thereby enhancing students' knowledge and skill acquisition.

## Introduction

Medical education comprises a diverse curriculum that necessitates continual updates in knowledge, technology, and instructional methods. Traditionally, instructors functioned as the primary source of information. Currently, there is a shift toward a learner-centric approach, particularly within competency-based medical education (CBME) [1]. In this context, "competency-based" refers to an educational focus on demonstrated abilities rather than time spent, while a "learner-centric" approach grants students greater autonomy over their learning processes. In CBME, teachers serve as facilitators, guiding and supporting learners rather than solely delivering information [1]. Traditional classroom instruction frequently lacks immediacy, rapid evaluation, and sufficient student engagement. Digital learning tools have been developed to address these challenges. With the increasing use of smartphones and wireless devices among students, educational institutions are integrating technology into classrooms to better engage contemporary learners. Ellaway and Masters characterise e-learning as flexible, engaging, and learner-centred. This approach promotes interaction and collaboration, often in asynchronous formats [2]. Digital learning enables students to control the timing, location, and pace of their learning, as well as the opportunity for repetition. E-learning employs visual and auditory modalities to clarify complex concepts [3]. Students can repeat tasks as needed, in identical or varied scenarios, to reinforce understanding. The United Nations and World Health Organisation endorse e-learning as a valuable resource for healthcare education, particularly in developing countries, underscoring its broad applicability and benefits [2].

However, there is limited research evaluating the retention of knowledge acquired through e-learning in both short- and long-term contexts. Most existing studies focus on Learning Management Systems (LMSs), leaving a significant research gap in the Indian context. The need for cost-effective, student-centred educational resources tailored to India's context is underscored by the country's allocation of 2.9% of its Gross Domestic Product (GDP) to medical education (Ministry of Finance, 2023) [4]. Studies indicate that proficiency improves with focused feedback, opportunities for repetition, and targeted practice, particularly in challenging areas [5,6]. Ongoing distributed practice is more effective at mitigating performance decline over time than single-session training [7]. Spaced repetition and test-enhanced learning have been demonstrated to improve recall, long-term retention, student satisfaction, self-directed learning, and higher-order reasoning among medical students [8,9]. Spaced repetition is a strategy in which learners review material multiple times, with progressively increasing intervals between reviews. In this context, spaced repetition refers to revisiting material after longer intervals. Conversely, massed educational interventions involve concentrated study over a brief period. Spaced repetition is more effective for long-term learning because it reinforces material at distributed intervals [5,8].

Test-enhanced learning refers to the phenomenon whereby testing, defined as answering questions on material after initial exposure, leads to greater retention than merely reviewing the material or employing techniques such as concept mapping. This effect is amplified by increased testing frequency, which means more frequent assessments and longer test intervals. Frequent testing through test-enhanced learning improves long-term memory retention and overall learning outcomes [5,9]. Studies have demonstrated that recall accuracy increases from 27% to 82% when utilising a study-quiz module incorporating spaced repetition and quizzes on a digital platform [6]. Furthermore, statistically significant improvements in teaching a traumatic restorative treatment to undergraduate and postgraduate students have been observed with the use of e-learning modules [9-11]. Various tools, like spaced-education games and emailed content, are used in medical education to deliver spaced learning. Blended teaching methods also show that spaced interventions help retention. The National Medical Commission (NMC) urges facilitators to develop online learning and assessment strategies [1,12]. The study aims to assess the effectiveness of digital learning as an adjunct to traditional instruction for MBBS Phase III Obstetrics and Gynaecology students. Specifically, the study measures the impact of digital learning on students’ ability to recall and retain knowledge.

## Materials and methods

This prospective, observational institute-based study was conducted from April to June 2024 at Heritage Institute of Medical Sciences, Varanasi, India. All MBBS Phase III Obstetrics and Gynaecology students were included, except those without internet access or digital tools. The digital tools utilised were Google Forms, email, and WhatsApp. Convenience sampling was employed to select 120 students. Structured digital modules featuring quiz-mode questions were implemented, and feedback was collected via Google Forms. Informed consent was obtained from all participants, and confidentiality and anonymity of responses were maintained.

All 120 students were taught one competency in OBGY (NMC curriculum) using the traditional didactic lecture method. Traditional learning consisted of two didactic lecture sessions, every 45 minutes. After the second lecture session, a predesigned, pre-validated structured Pre-test was administered to all 120 students via Google Forms. Following the pre-test, students were divided into two groups of 60 each based on their odd or even roll numbers. Students with odd roll numbers formed the traditional learning group (T), while those with even roll numbers comprised the digital learning group (D). A digital learning session was conducted for group (D), which received a Google Forms quiz on the same competency in Obstetrics and Gynaecology. The quiz presented one question at a time, and students could proceed to the next question only after answering correctly (active education). Each correct answer was awarded one mark.

Group (T) continued their learning independently, following the institutional curriculum. A post-test was administered to both groups (T and D) after the learning session, consisting of 20 questions, each worth one mark. Feedback on digital learning was collected from group D with Google Forms, using closed questions and a Likert scale. Participants were not informed about the pre- and post-tests in order to prevent preparation for test and reduce bias. Data were collected, tabulated, and statistically analysed using Microsoft Excel and IBM SPSS Statistics for Windows, Version 25 (Released 2017; IBM Corp., Armonk, New York, United States). An unpaired Student's t-test was employed to analyse the results. A p-value < 0.05 was considered statistically significant. Knowledge and digital media effectiveness were evaluated by comparing group pre- and post-test results. Knowledge retention was assessed by analysing pre- and post-test scores. Perceptions of digital learning were assessed using feedback forms from group D.

## Results

One hundred twenty MBBS Phase III students were enrolled in the study. It consisted of 55% (n=66) male students & 45% (n=54) female students. Among them, 61.7% (n=74) of the students were in the age group of 23-24 years, while 20% (n=24) were between 21 and 22years, and 18.3% (n=22) were above 25 years of age. Comparing the two groups, the pre-test mean score was 16 in both groups, but the post-test scores varied: the mean increased to 17.33 in Group D and decreased to 15.97 in Group T, as shown in Table 1. The variations in scores were seen in the pre-test of both groups (SD=2.8). But group T post-scores continued to vary, with a minimum of 7 and a maximum of 20 (SD=2.5), whereas group D showed less variation, with a minimum of 12 and a maximum of 20 (SD=1.7), as shown in Table 1.

**Table 1:** Pre-test and post-test scores of group (D) and group (T)

Statistics	Group D Students (N=60) Pre-score	Group D Students (N=60) Post-score	Group T Students (N=60) Pre-score	Group T Students (N=60) Post-score
Mean score	16	17.33	16	15.97
Median	17	18	17	16
Std. Deviation	2.846	1.791	2.882	2.558
Minimum score	8	12	7	7
Maximum score	20	20	20	20
25th Percentiles	14	16.25	14.25	14
50th Percentiles	17	18	17	16
75th Percentiles	18	18	18	18

The results showed that when pre-test and post-test scores were compared using a t-test for group D, it was found to be statistically significant (p=0.002) (p <0.05 considered significant) as shown in Table 2.

**Table 2:** Comparison of pre- and post-test scores for each individual group *p< 0.05 considered statistically significant

Paired Samples Test	Paired Samples Test	Paired Differences (Mean)	Paired Differences (Std. Deviation)	Paired Differences (Std. Error Mean)	Paired Differences (95% Confidence Interval- Lower)	Paired Differences (95% Confidence Interval- Upper)	t stats	Degrees of freedom	p-value
Group D	Pre-score - post-score	-1.333	3.240	.418	-2.170	-.496	-3.188	59	.002*
Group T	Pre-score - post-score	.033	2.986	.385	-.738	.805	.086	59	0.931

**Figure 1:**
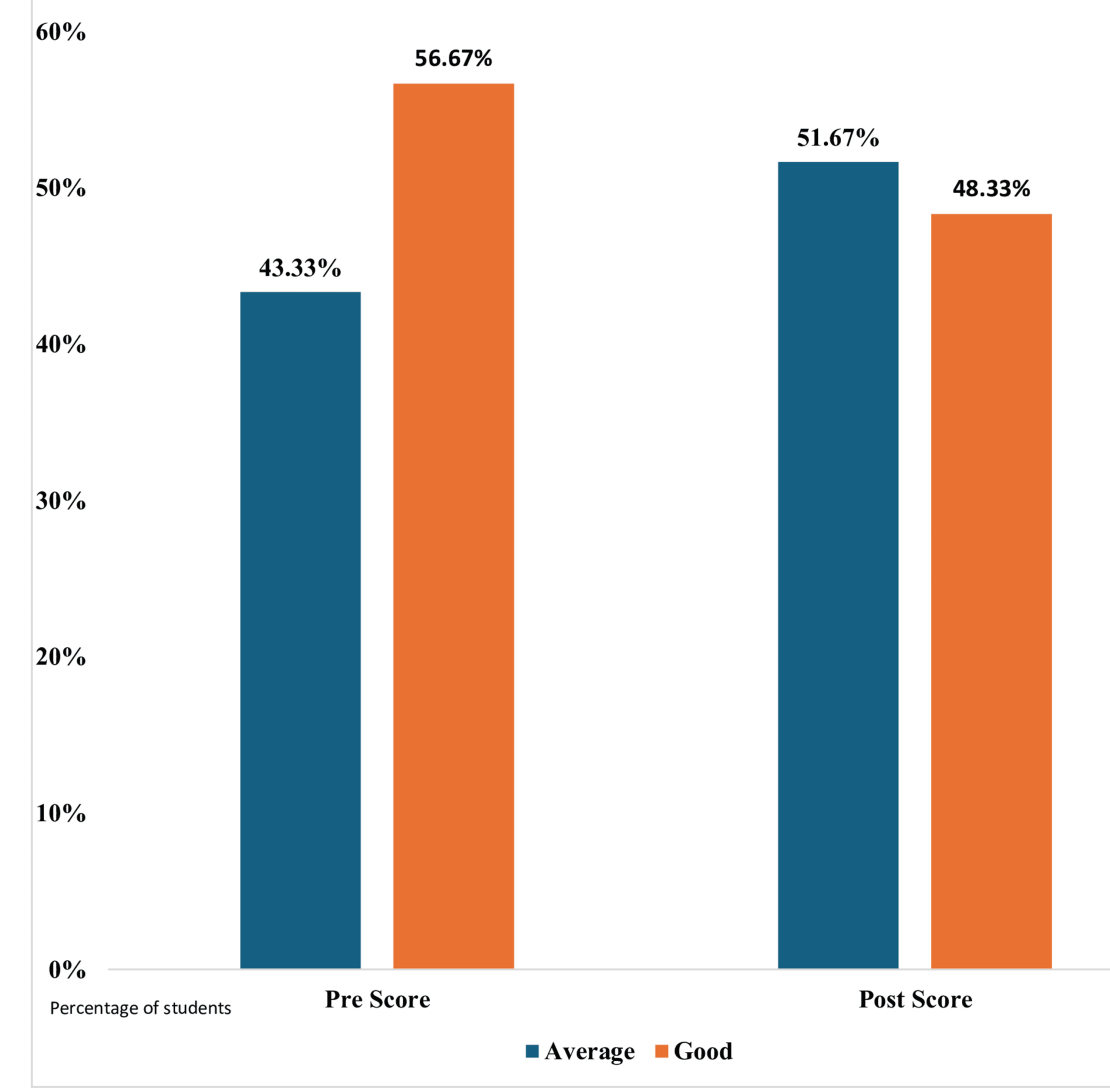
Comparison of pre- and post-test scores of group T

**Figure 2:**
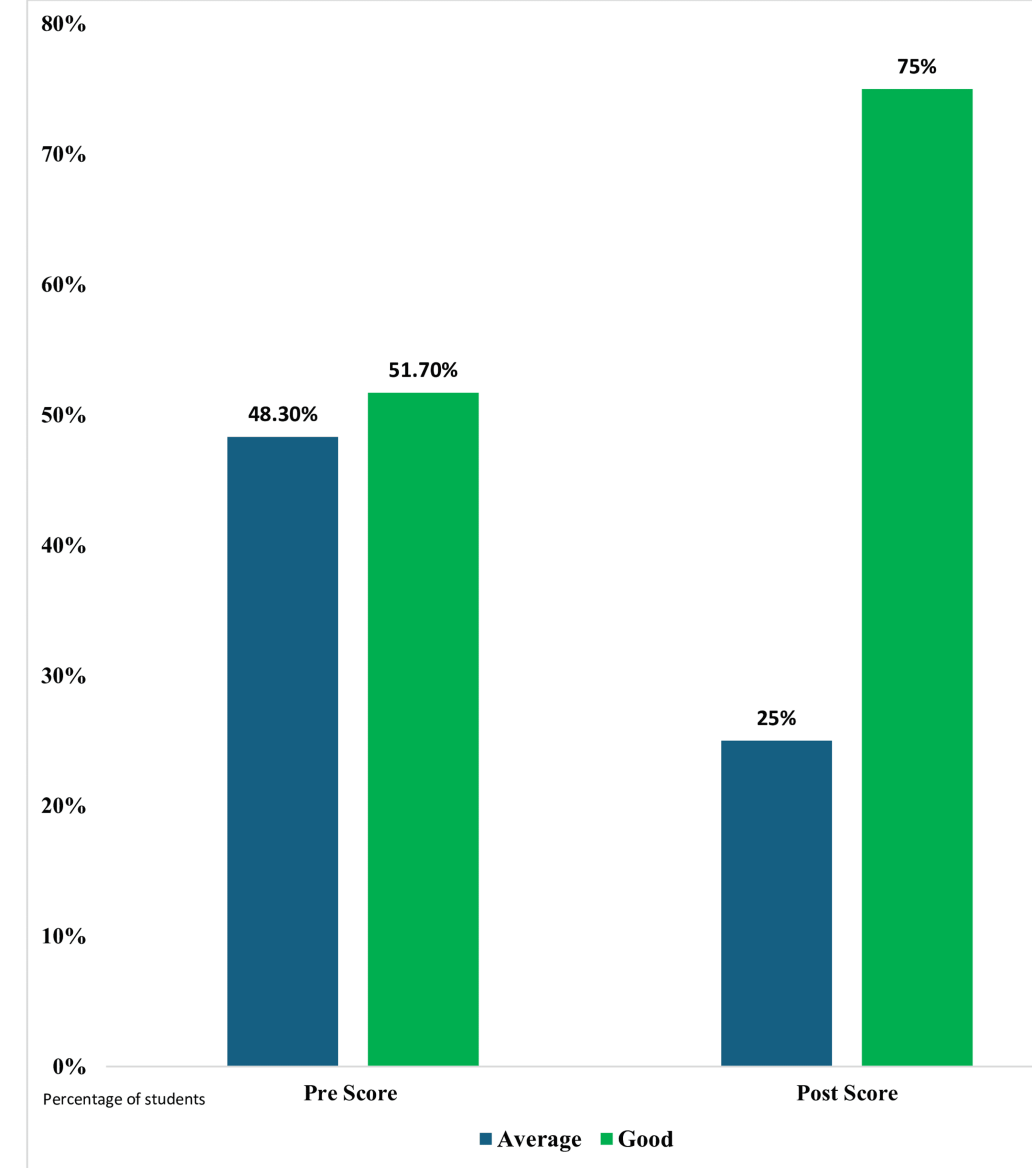
Comparison of pre- and post-test scores of group D

Upon microanalysis of the results, in which students’ scores were categorised into 16 (average score) and 17 (good score), the following results were obtained. The pre-test results of both groups were similar: group T and group D students scoring an average of 16 were 43.33% and 48.3% of students, respectively, while good scores (17) were attained by 56.67% of students in group T and 51.70% in group D in the pre-test. The post-test results for group T students showed a decline in students scoring good (17) from 56.67% to 48.33%, and an increase in students scoring average (16) from 43.33% to 51.67%. While the post-test scores of group D showed a higher number of students (75%) achieving good scores (17), the average score (16) was reduced to just 25% of students, as shown in Figures 1, 2.

When students were asked about what they liked in this digital module, their responses are shown in Table 3.

**Table 3:** Positive feedback of group D students about the digital module MCQ: Multiple-choice question

Sr. No.	Responses of Group D Students on What They Liked About the Digital Module
1.	A realistic experience of giving exams in a simulation. An excellent effort at making us accustomed to taking tests, thus diminishing our fear of tests. The questions were also on topics that were well discussed in lectures. An overall essential attempt to make understanding easier
2.	We can use it anytime and are excellent mode to test our memories.
3.	It helps to retain knowledge that is being taught in the classroom.
4.	The topic gets revised. Revision and practice for this topic.
5.	By attending a virtual escape room, I got to know where I have doubts and where I am going wrong. Secondly, solving clinical questions.
6.	Help to recap the lectures. Good for evaluating ourselves.
7.	It helps me to gain confidence after solving the Google Form.
8.	It helped me build a better understanding of the topics/content to focus on while reading. Also helped in the conceptual application of facts we learnt with a topic-centric approach.
9.	Interesting and engaging.
10.	More frequent. Small topics at once. Only MCQ.
11.	This method is very helpful, although making it a bit user-friendly will make a lot of difference.

The feedback on what the students disliked and faced difficulty in the digital module was mostly on technical issues, where the digital module “required answers in fixed syntax & was not accepting responses if it varied”, “had network issues”, login -in issues”, “surprise test”, “quiz mode”. Still, 91.7% (n=55) of students wanted this type of digital module in the future to enhance their learning, 6.7% (n=4) were unsure, and 1.7% (n=1) did not want it. 86% (n=51) of students were satisfied with the digital module.

## Discussion

Digital learning, or e-learning, is one of the most promising tools for learning in the modern era, adapted to society's needs. It is being avidly used in schools and colleges. Digital learning helps students focus and concentrate for longer periods than traditional teaching methods [3]. Audiovisual sessions, escape rooms and coloured slides promote interactive learning, and long-lasting results are seen. Students tend to grasp easily due to the ease of access, flexibility of time and place, interactivity, and personalised experience; all of which make e-learning capable of augmenting traditional learning [6]. 

The effectiveness of e-learning on retention and recall among MBBS Phase III students was evaluated in this study. A total of 120 students participated, all of whom attended a lecture on Obstetrics and Gynaecology. Of these, 60 students received a digital learning module (group D), while the remaining 60 students continued with traditional learning (group T). The cohort comprised 66 (55%) male and 54 (45%) female students. The majority (61.7%) were aged 23-24 years, 20% were between 21 and 22 years, and 18.3% were above 25 years of age. The pre-test results indicated minimum scores of 7 and 8 for groups T and D, respectively, with a maximum score of 20 in both groups. In the post-test, the minimum score remained 7 for group T but increased substantially to 12 for group D, suggesting that lower-performing students in group D improved following digital learning. These findings align with those of Duraes et al. (2023), who observed a significant increase in overall knowledge (10.6%, p = 0.09) in their e-learning group [10]. The pre-test scores of group D improved significantly after the digital module (p=0.002), whereas improvement in group T was not statistically significant (p=0.931), indicating a decline in recall over time without digital support. Similarly, Taveira-Gomes et al. reported a recall accuracy of 91% in the quiz group using a digital platform among medical students, consistent with the present results [5].

When students were categorised as average performers (score <16) and good performers, analysis revealed that pre- and post-intervention scores in group D were statistically significant (p<0.016), whereas group T did not show a significant change (p=0.405). In the average-scoring category (<16), 43.33% of group T and 48.3% of group D students fell into this range on the pre-test. Scores above 17 were achieved by 56.67% of group T and 51.70% of group D students in the pre-test. However, the proportion of students scoring above 17 declined in group T and increased to 75% in group D after the intervention. These findings indicate that average-performing students in group D improved and moved into the good-performing category, while the control group declined in performance. Therefore, digital modules benefit both high- and low-performing students by enhancing knowledge and outcomes. These results are consistent with those of Camargo et al., who studied 78 dental students using e-learning (p<0.001) [9], and with findings by Raupach et al. among 183 third-year MBBS students learning a cardiorespiratory curriculum using digital media (84.8 ± 1.3 vs.79.5 ± 1.4%; p = 0.006; effect size 0.67) [11].

A study done by Stecula and Wolniak, including 621 students to know the advantages and disadvantages of innovative e-learning during the COVID-19 pandemic, concluded that e-learning is advantageous in ease of acquiring content and the students who assimilate innovative e-learning technology easily, the higher the student evaluates it as advantageous [13]. In the study conducted by Murphy et al. (2023), it was found that if students choose to engage in massed studying (by virtue of constraints on their study time or a failure to appreciate the benefits of spaced study sessions) [8], then studying the information twice but for half the time may produce memory benefits in a single study session. Thus, time spent on studying is important for recall and retention.

When asked about the module's likes and dislikes, most responses were positive. 91.7% of the students want more future sessions like this digital module in the future. Reasons for liking this learning were help with retention and recall of the topic, quiz mode that makes it seem playful and appealing, clarity of concepts, being less time-consuming, giving them confidence to prepare for exams, and providing an explanation after each question. The reasons for dislike (13.4%) of this module were mainly technical issues, which could be better addressed with support from the institution and the IT department. Various types of questions can be prepared, ranging from MCQs and short-answer questions to video-based, clinical questions, and problem-based learning. The dislikes were solely related to technical issues. 

Digital learning, characterised by effective communication, interaction, accessibility, acceptance, and low cost, represents a powerful educational tool [14,15]. In India, where medical institutions serve a large and diverse student population, curricula must be adapted to local requirements, including students’ needs and perceptions, limited human resources, IT infrastructure, and overloaded curricula. Modules such as the one studied can help bridge these gaps. Retention, recall, and application of knowledge are fundamental to effective learning. The use of digital tools that incorporate spaced repetition and testing can help organise students' learning, provide real-time, personalised feedback, manage future study sessions, and promote self-directed learning [16,17]. However, certain limitations exist, such as sample size and being a monocentric study. Also, some students and teachers who are not proficient with digital devices may be reluctant to adopt this new teaching and learning method. Further research involving larger student cohorts and a meta-analysis on this topic involving students from different institutes across India is necessary to provide additional insights into this topic. Sample size and a unicentric study were also limitations. There are a few other challenges such as computer vision syndrome, dry eye disease, and eye irritation, which present obstacles to large-scale implementation [18].

## Conclusions

This module appears to address gaps for students who rely exclusively on traditional learning methods. It enhances knowledge and retention, which are critical for success in the extensive medical curriculum. The module enables spaced repetition, which supports knowledge retention and reduces exam-related anxiety. Its asynchronous format accommodates large student cohorts while providing personalised feedback, offering advantages over traditional methods. The accessibility in terms of time and location makes it appealing to both students and teachers. Although initial development is time-intensive, the module can be utilised in low-resource settings and, with institutional support, expanded into LMSs.

## Figures and Tables

**Figure 3:**
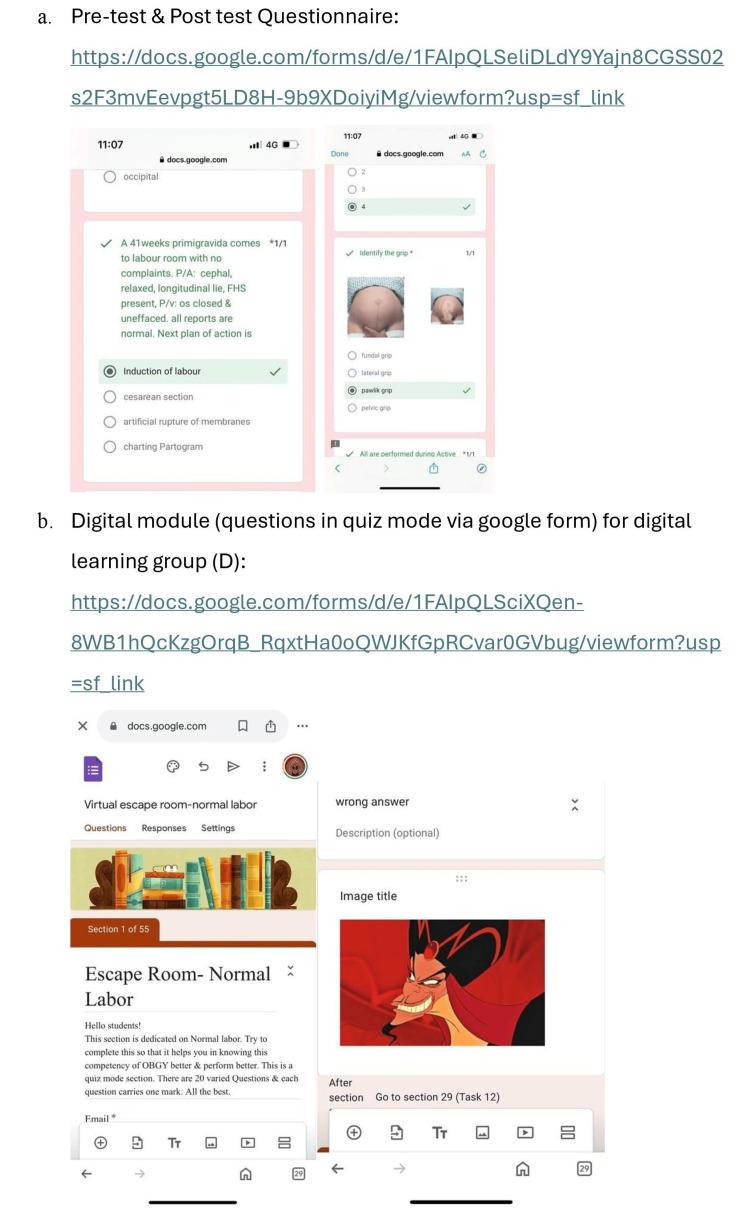
Snapshot of a part of the pre-test and post-test questionnaire

**Figure 4:**
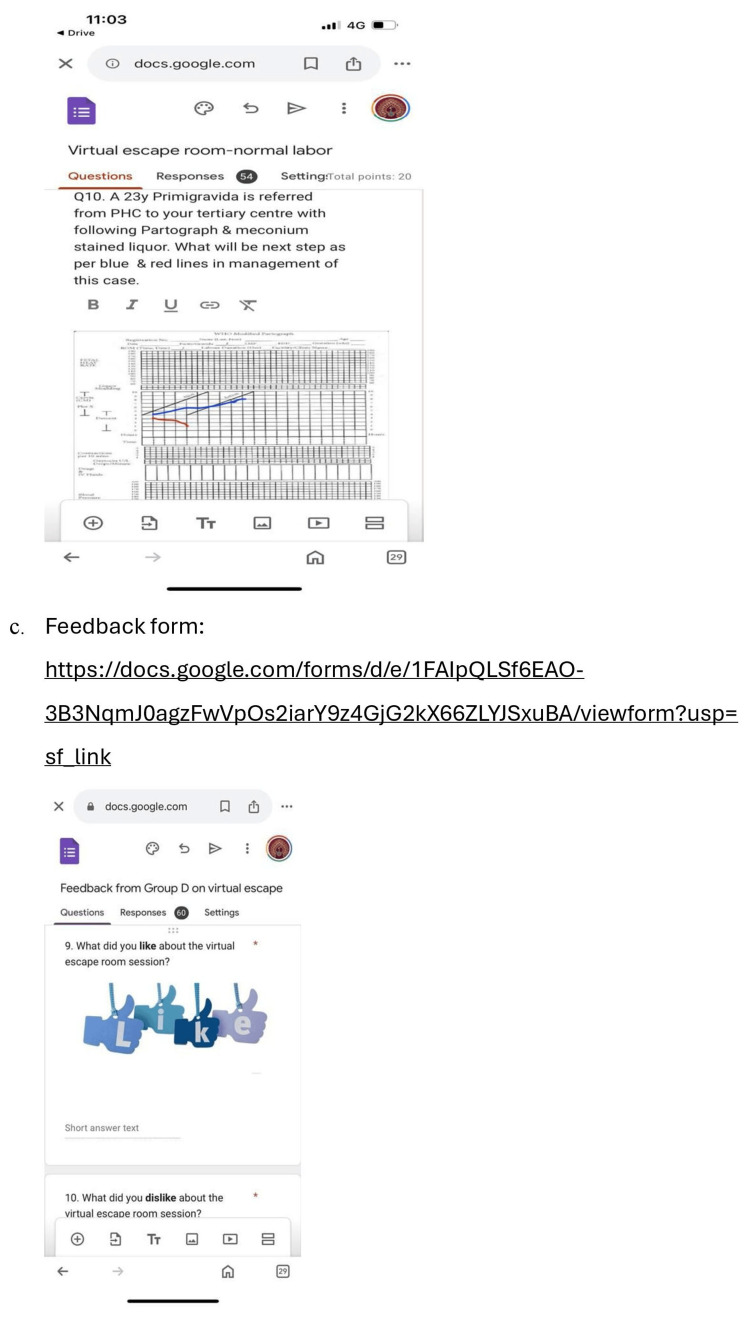
Snapshot of a part of feedback form

## Appendices

Pre-test and post-test questionnaire presented to the students

https://docs.google.com/forms/d/e/1FAIpQLSeliDLdY9Yajn8CGSS02s2F3mvEevpgt5LD8H-9b9XDoiyiMg/viewform?usp=sf_link

Digital module (questions in quiz mode via Google Form) for digital learning group (D)

https://docs.google.com/forms/d/e/1FAIpQLSciXQen-8WB1hQcKzgOrqB_RqxtHa0oQWJKfGpRCvar0GVbug/viewform?usp=sf_link

Feedback form

https://docs.google.com/forms/d/e/1FAIpQLSf6EAO-3B3NqmJ0agzFwVpOs2iarY9z4GjG2kX66ZLYJSxuBA/viewform?usp=sf_link
